# Flexural Stiffness of Myosin Va Subdomains as Measured from Tethered Particle Motion

**DOI:** 10.1155/2015/465693

**Published:** 2015-11-30

**Authors:** Arthur J. Michalek, Guy G. Kennedy, David M. Warshaw, M. Yusuf Ali

**Affiliations:** ^1^Department of Molecular Physiology and Biophysics, University of Vermont, Burlington, VT 05405, USA; ^2^Department of Mechanical & Aeronautical Engineering, Clarkson University, Potsdam, NY 13699, USA

## Abstract

Myosin Va (MyoVa) is a processive molecular motor involved in intracellular cargo transport on the actin cytoskeleton. The motor's processivity and ability to navigate actin intersections are believed to be governed by the stiffness of various parts of the motor's structure. Specifically, changes in calcium may regulate motor processivity by altering the motor's lever arm stiffness and thus its interhead communication. In order to measure the flexural stiffness of MyoVa subdomains, we use tethered particle microscopy, which relates the Brownian motion of fluorescent quantum dots, which are attached to various single- and double-headed MyoVa constructs bound to actin in rigor, to the motor's flexural stiffness. Based on these measurements, the MyoVa lever arm and coiled-coil rod domain have comparable flexural stiffness (0.034 pN/nm). Upon addition of calcium, the lever arm stiffness is reduced 40% as a result of calmodulins potentially dissociating from the lever arm. In addition, the flexural stiffness of the full-length MyoVa construct is an order of magnitude less stiff than both a single lever arm and the coiled-coil rod. This suggests that the MyoVa lever arm-rod junction provides a flexible hinge that would allow the motor to maneuver cargo through the complex intracellular actin network.

## 1. Introduction

Myosin Va (MyoVa) is a processive molecular motor [1] involved in intracellular cargo transport along the actin cytoskeleton [2, 3]. Each dimerized molecule consists of a pair of N-terminal motor domains (i.e., heads) connected to lever arms, followed by a coiled-coil rod domain, ending in a globular C-terminal cargo-binding domain [4]. MyoVa's directed movement is accomplished by a conformational change in the motor domain following ATP hydrolysis [5, 6]. This motion is amplified by the lever arms, resulting in the motor's processive motion as each head takes 72 nm steps in a hand-over-hand fashion [7–9].

Each lever arm consists of six tandem *α*-helix motifs, stabilized by calmodulin or calmodulin-like light chains [10]. Calcium is required to activate the ATPase activity of MyoVa [11]; however in the presence of high calcium (>1 *μ*M), motor processivity is reduced [12] and one or more calmodulins may dissociate from each lever arm [13]. Calmodulin dissociation may render the lever arm highly compliant, which could reduce the efficiency of both the lever arm displacement amplification and the strain-dependent gating between the two heads, which are necessary for the motor's processivity.

Single MyoVa are adept at maneuvering through actin intersections and traveling along actin bundles in vitro [14–17]. MyoVa must have sufficient structural flexibility to permit the unbound leading head to undergo a broad diffusive search [7, 18–20], allowing MyoVa to switch actin tracks even when the angle between intersecting actin filaments is ~150° [14]. The structural domains that are critical to MyoVa's maneuverability have yet to be determined but may reside within the lever arms or at the lever arm/coiled-coil junction [21].

**Figure 1:**
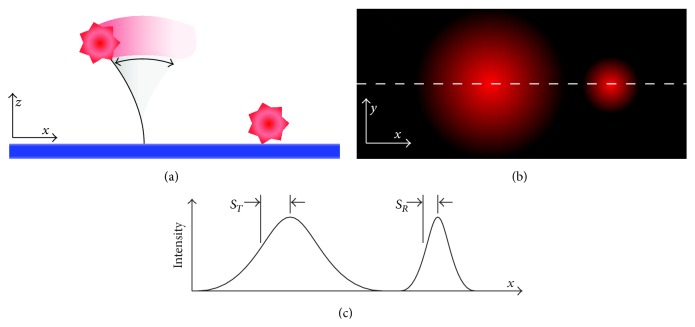
Tethered Qdot motion. Brownian motion causes a Qdot (red hexagon) fixed to an elastic tether to move (a), resulting in an increase in apparent point spread function (b). In both the tethered and surface bound states, the point spread functions are Gaussian in shape (profile along dashed line shown in (b), with standard deviations corresponding to image size parameters *S*_*T*_ and *S*_*R*_, respectively.

**Figure 2:**
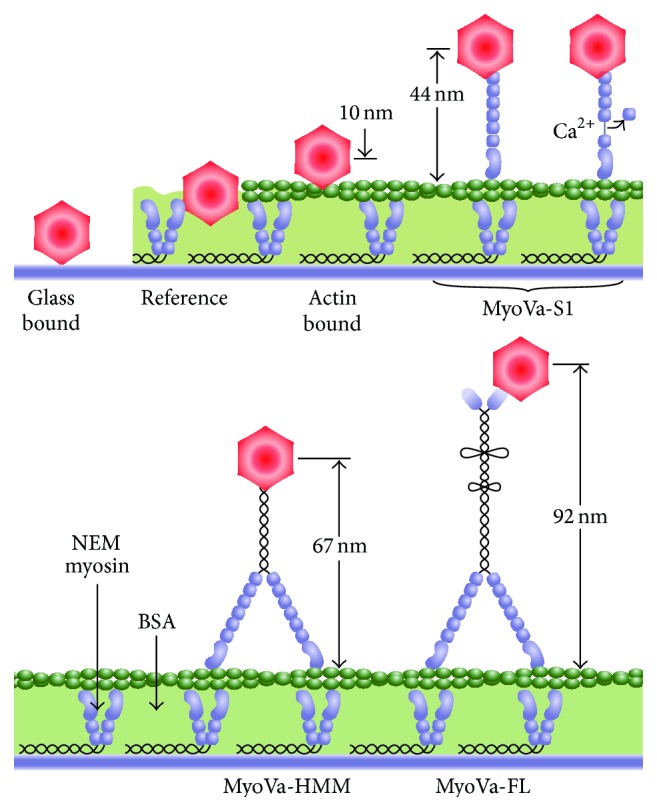
Summary of MyoVa constructs and Qdot (red hexagon) labels. Lengths of MyoVa constructs [27] are estimated from the actin filament to the center of the Qdot. Overall length of MyoVa-FL was estimated based on the average value of reported distal tail lengths [4, 27].

Here we used tethered particle microscopy (TPM) to directly measure the flexural stiffness of individual MyoVa motors and their subdomains. TPM has been used extensively to characterize the contour length of DNA tethers [22–25]. TPM has additionally been coupled with Monte Carlo simulations of nanoparticles attached to wormlike chain tethers to estimate, with high precision, the persistence length (a measure of flexural stiffness) of double-stranded DNA [26]. Our utilization of TPM involved anchoring a MyoVa-actin complex to a microscope slide and then attaching a visible particle (i.e., fluorescent quantum dot (Qdot)) to a desired location along the motor. Thermal energy caused the Qdot to be randomly displaced within bounds defined by the length and stiffness of the effective tether (i.e., the MyoVa). The resulting motion was measured by comparing the difference in apparent image size of the tethered Qdot to a stationary, reference Qdot, both of which are viewed over a long (~2.5 s) exposure time [24, 25]. While a Qdot held in a fixed location has an apparent image size (*S*_*R*_) which is dependent on its diffraction limited point spread function, tethered Qdots will appear larger (*S*_*T*_) depending on the length and flexibility of the tether (Figure 1). By attaching Qdots to expressed MyoVa constructs of different lengths (Figure 2), we isolated the contributions of individual structural domains, for example, the lever arm and rod domain, to the overall flexibility and flexural stiffness of the MyoVa molecule. In addition, we hypothesized that calmodulin disassociation from the MyoVa lever arm decreases the lever arm's flexural stiffness, thus contributing to the motor's compromised processivity in the presence of calcium.

## 2. Materials and Methods

### 2.1. Protein Expression and Purification

Various MyoVa constructs, listed as follows, were expressed in the* Baculovirus*/Sf9 cell system and purified as described previously [11]. These constructs allowed the flexural stiffness of MyoVa subdomains to be defined by TPM (Figure 2). Full-length murine MyoVa (MyoVa-FL) was expressed as was a double-headed short construct (MyoVa-HMM), engineered by truncating the MyoVa-FL heavy chain at residue 1098, followed by a biotin tag and a FLAG epitope tag at the C-terminus to facilitate purification [14, 28]. Double-headed MyoVa constructs were coexpressed with calcium-insensitive calmodulin (CamΔall) as described previously [11]. Single-headed MyoVa (MyoVa-S1) was truncated at amino acid 908 with a biotin tag at the C-terminal end and coexpressed with wild-type calmodulin and purified as described [29]. The MyoVa-S1 and its wild-type calmodulins are sensitive to calcium in that calmodulin(s) will dissociate from the lever arm in the presence of calcium in the solution [12]. The biotin tag is an 88-amino-acid segment from the* Escherichia coli* biotin carboxyl carrier protein [30], which was biotinylated at a single lysine residue (located 35 amino acids from the C-terminus) during expression in* Sf9* cells. N-Ethylmaleimide- (NEM-) modified skeletal muscle myosin was prepared as previously described [31] and was used to strongly adhere actin filaments to the glass surface. Actin filaments were isolated from chicken pectoralis; labeled with tetramethyl rhodamine isothiocyanate (TRITC) phalloidin overnight in Buffer A (25 mM imidazole, pH 7.4, 4 mM MgCl_2_, 1 mM EGTA, 25 mM KCl, and 10 mM DTT) prior to experiments.

### 2.2. Qdot Attachment to MyoVa Constructs and Actin Filaments

Carboxylated Qdots, emitting at 655 nm (Invitrogen-Molecular Probe, Eugene, OR), were attached to the MyoVa-FL cargo-binding domain by incubating a 4 : 1 mixture of Qdots to MyoVa-FL for 15 min on ice in Buffer B (25 mM imidazole, pH 7.4, 4 mM MgCl_2_, 1 mM EGTA, 25 mM KCl, 10 mM DTT, oxygen scavenger composed of 0.1 mg/mL glucose oxidase, 0.02–0.18 mg/mL catalase, and 3.0 mg/mL glucose) and 0.1 mg/mL BSA [32]. Streptavidin-conjugated Qdots, also emitting at 655 nm (Invitrogen-Molecular Probe, Eugene, OR), were attached to the C-termini of the MyoVa-HMM and MyoVa-S1 constructs by mixing Qdots and construct (4 : 1 ratio) and incubating for 15 minutes on ice in Buffer B plus 0.5 mg/mL BSA [32]. The mixture was further diluted to a final MyoVa construct concentration of ~0.1 nM before imaging. To attach streptavidin-conjugated Qdots to actin filaments, biotinylated actin filaments were prepared by mixing 1 *µ*M actin filament, 500 nM TRITC-phalloidin, and 500 nM biotin-phalloidin (Invitrogen) in Buffer A and then incubating overnight at 4°C. Biotinylated actin filaments (50 nM) were attached to the glass surface through NEM-modified myosin (see Section 2.3 for details). After washing the flow cell with Buffer A, 0.5 mg/mL BSA was added. Then 2–4 nM streptavidin-conjugated Qdots (emitting at 655 nm) were introduced into the flow cell. To remove the unbound Qdots, the flow cell was washed by Buffer A.

### 2.3. Single Molecule Assay

A 20 *µ*L flow cell was constructed as described [28]. First, 20 *µ*L of NEM-modified myosin [8] at 0.5 mg/mL in Buffer C (25 mM imidazole, pH 7.4, 4 mM MgCl_2_, 1 mM EGTA, 300 mM KCl, and 10 mM DTT) was introduced and incubated for 2 minutes at room temperature. NEM-modified myosin was used to fix the actin filaments to the glass surface, as it has strong binding affinity to actin. After washing the flow cell using Buffer B, 20 *µ*L of 0.5 mg/mL BSA in Buffer B was infused and incubated for 2 min, followed by a Buffer B wash. TRITC-phalloidin labeled actin in Buffer B was then introduced into the flow-cell chamber and incubated for 2 min, followed by a Buffer B wash containing a MyoVa:Qdot complex. All MyoVa constructs were strongly bound to actin in the absence of MgATP, that is, in rigor. It is assumed that MyoVa constructs bound to the top surface of the actin filaments due to the high entropy required to approach the filaments from the sides [33]. To measure the calcium-dependent flexibility of the MyoVa-S1 construct, calcium was added to the MyoVa-S1:Qdot complex in Buffer B, to a final concentration of 1 *μ*M [12], and then introduced into the flow cell.

### 2.4. Data Acquisition, Image, and Tethered Particle Motion Data Analysis

Single molecule imaging was performed at room temperature (23 ± 1°C) using a Nikon Eclipse Ti-U inverted microscope equipped with a PlanApo objective lens (100x, 1.49 n.a.) through the objective TIRF microscopy. Images were acquired using PIPER software (Piper control v2.3.14 software, Stanford Photonics, Stanford, CA) and a high resolution (95 nm per pixel) digital camera (Standard Photonics, Turbo-Z, Stanford, CA). Qdots were excited with a 473 nm argon laser line and images obtained using a 655 ± 20 nm emission filter (Chroma Technologies, Rockingham, VT) at 8.3 ms integration time for 300 frames. Following imaging of the Qdots, a single image of the actin filaments in the same visual field was obtained by exciting the TRITC-phalloidin labeled actin with a 532 nm laser line and the emission acquired through a 605 ± 35 nm filter (Chroma Technologies, Rockingham, VT) and a 67 ms exposure time.

**Figure 3:**
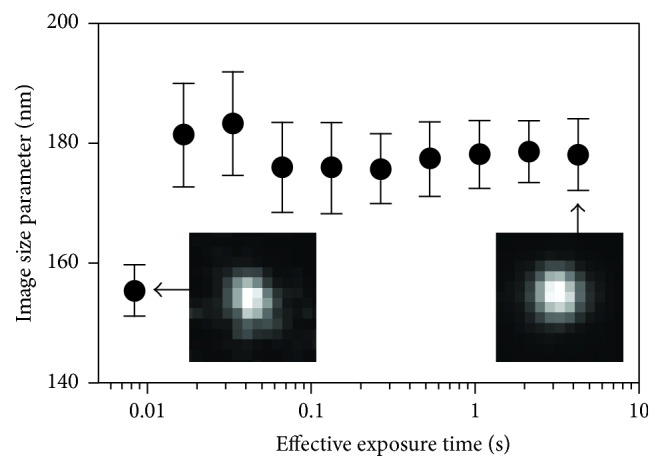
Image size parameter versus effective camera exposure time. The number of frames necessary to sample the full extent of Qdot motion was established by summing increasing numbers of 8.3 ms exposures of 33 Qdots tethered by full-length MyoVa. Lower effective exposure times tended to underestimate the image size parameter (shown as median ± standard error of the median as calculated by (1) in text). The effective exposure time used for analysis (2.49 s) was approximately ten times longer than that required to reach a point where the image size parameter was no longer changing. Inserts show the same Qdot imaged for one frame (left) and for the full 2.49 s integration (right).

**Figure 4:**
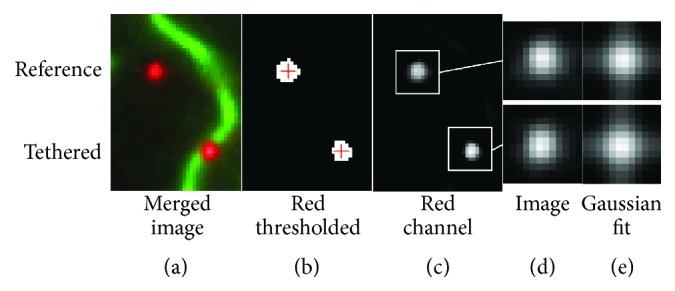
Protocol for identifying and fitting reference and tethered Qdot images. Qdots bound to the NEM-myosin surface (reference) and tethered by full-length MyoVa in this example (tethered) (a) were located by thresholding the Qdot image channel and identifying continuous, round regions (b). The centers of these regions were used to locate 14 pixel square subimages (white squares in (c)). These subimages were then extracted (d) and fit with a two-dimensional Gaussian function (e) providing image size parameters: *S*_*T*_ = 168.1 nm and *S*_*R*_ = 165.3 nm.

Images were analyzed using a set of custom written Matlab (Mathworks Inc., Natick, MA) routines. First, each series of frames was converted to double precision and summed for an effective integration time of 2.49 s, which was determined to be suitably long to sample the full extent of particle motion (see Results and Discussion; Figure 3). Summation of short exposures was chosen for this study rather than a single long exposure in order to reduce noise and confirm that the microscope stage did not drift during the experimental protocol. Qdots were automatically identified (Figures 4(a) and 4(b)) by thresholding the summed images to binary and recording the centroids of regions which fit three different selection criteria. Regions were included based on size (<144 pixels^2^) and elliptical eccentricity (<0.86, which is an aspect ratio of approximately 2 : 1) to reject regions likely representing multiple Qdots in close proximity to each other. Additionally, distance from edges of the image was required to be at least 12 pixels. Qdot apparent image sizes were then measured by extracting a twelve-pixel square subwindow around each Qdot (Figures 4(c) and 4(d)). This window size was suitably large to fully encompass the Qdot image that on average was approximately seven pixels in diameter. Each window was then fit, using the least squares method, by a two-dimensional Gaussian function (Figure 4(e)), in local coordinates *x*-*y* of the form

(1) G=A12πσx2e−x+Δx2/2σx212πσy2e−y+Δy2/2σy2+B,

where *A* is the intensity of the Qdot, *B* is the mean intensity of the background, *σ*_*x*_ and *σ*_*y*_ are the *x*-axis and *y*-axis standard deviations of the Gaussian function, and Δ*x* and Δ*y* are offsets between the center of the two-dimensional Gaussian function and the center of the window. An image size parameter, *S*, for each Qdot was then defined as the average of *σ*_*x*_ and *σ*_*y*_ multiplied by a microscope image calibration factor (95 nm per pixel). Surface bound Qdots (i.e., stationary and fixed) were used as an internal reference and control for the tethered Qdot image size parameters in the same field of view, whether they be on an actin filament or a MyoVa construct. The reference and tethered Qdots were differentiated by merging images of Qdots and actin filaments and manually selecting the Qdots not colocalized with actin as reference Qdots (Figure 4(a)).

When two orthogonal (i.e., not correlated) signals oscillating around a central point are summed, the resulting variance is the sum of the variances of the two signals. Given two signals with standard deviations *σ*_1_ and *σ*_2_, the sum of the two signals will thus have a deviation, *σ*_3_, given by the square root of the sum of squares:

(2) $$\begin{array}{c} \sigma_{3}=\sqrt{\sigma_{1}^{2}+\sigma_{2}^{2}}. \end{array}$$

**Figure 5:**
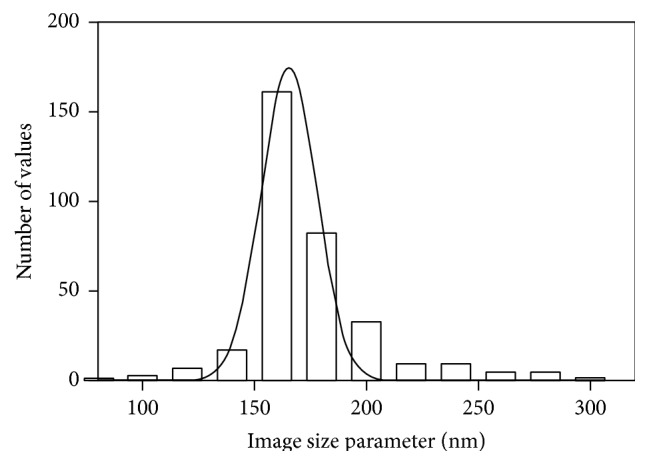
Histogram of image size parameters for Qdots tethered by full-length MyoVa (median = 168.34 nm). Solid line indicates a Gaussian fit in order to emphasize the heavy tail of the distribution and justify the use of the median to characterize the image size parameter.

Both the reference image size parameter, *S*_*R*(*∗*)_, and the tethered particle motion, *δ*_*T*(*∗*)_ (where *∗* may refer to images with Qdots bound to actin filaments, *A*, or MyoVa constructs, *M*), are defined as the deviations of normally distributed random signals, which, added together, result in an apparent image size parameter for the tethered Qdot (i.e., *S*_*T*(*∗*)_). Therefore, *S*_*T*(*∗*)_ results from letting *σ*_1_ = *S*_*R*(*∗*)_ and *σ*_2_ = *δ*_*T*(*∗*)_ in (2). This then allows the motion of the tethered particle to be determined when median values of reference (*S*_*R*(*∗*)_) and tethered Qdot (*S*_*T*(*∗*)_) image size parameters are known as in (3). Due to nonnormality of image size parameter frequency distributions (Figure 5), median proved to be a more appropriate measure than the mean

(3) ST∗=SR∗2+δT∗2,δT∗=ST∗2−SR∗2.

Equation (2) may also be used to relate the deviations of any two points on a randomly oscillating structure that is fixed at one end to a rigid structure. This is particularly useful for canceling out the compliance of the NEM-modified myosin, which results in motion of the actin filaments, *δ*_*T*(*A*)_. The motion of a Qdot tethered by a MyoVa construct, corrected for actin filament motion, *δ*_*MA*_, may then be determined knowing the myosin tethered Qdot motion, *δ*_*T*(*M*)_, and the baseline actin filament motion, *δ*_*T*(*A*)_:

(4) $$\begin{array}{c} \delta_{MA}=\sqrt{\delta_{T\left(M\right)}^{2}-\delta_{T\left(A\right)}^{2}}. \end{array}$$

By substituting (3) into (4), the motion of a particle tethered by a MyoVa construct relative to actin, *δ*_*MA*_, was related to the various median measured image size parameters:

(5) $$\begin{array}{c} \delta_{MA}=\sqrt{S_{T\left(M\right)}^{2}-S_{R\left(M\right)}^{2}-S_{T\left(A\right)}^{2}+S_{R\left(A\right)}^{2}}. \end{array}$$

For median values of *S*_*T*(*∗*)_ and *S*_*R*(*∗*)_ measured for each construct, the standard error of the median (SEM) was calculated from the standard deviation (SD) and sample size (*n*) assuming an approximately Gaussian distribution:

(6) $$\begin{array}{c} \text{SEM}=\frac{1.25\text{SD}}{\sqrt{n}}. \end{array}$$

Error propagation through (5) (*E*_*δMA*_) was calculated from the median image size parameters of tethered and actin-bound Qdots (*S*_*T*(*M*)_ and *S*_*T*(*A*)_) along with the sizes of their associated reference Qdots (*S*_*R*(*M*)_ and *S*_*R*(*A*)_) and their respective standard errors (*E*_*T*(*M*)_, *E*_*T*(*A*)_, *E*_*R*(*M*)_, and *E*_*R*(*A*)_):

(7) EδMA∂δTA∂STM2ETM2+∂δTA∂SRM2ERM2+∂δTA∂STA2ETA2+∂δTA∂SRA2ERA2=STM2ETM2+SRM2ERM2−STA2ETA2+SRA2ERA2STM2−SRM2−STA2+SRA2.

The flexural stiffness for the various MyoVa constructs, *k*, was determined by equipartition due to thermal energy, resulting in Brownian motion of the tethered Qdot relative to the actin filament, *δ*_*MA*_, where *k*_*b*_ is Boltzmann's constant and *T* is temperature in degrees Kelvin:

(8) $$\begin{array}{c} k=\frac{k_{b}T}{\delta_{MA}^{2}}. \end{array}$$

The persistence length of the tether, *L*_*P*_, is defined as *EI*/*k*_*b*_ *T*, where *EI* is the flexural rigidity of a prismatic rod. Assuming that the tether acts as a cantilevered beam, with load imparted primarily through the Qdot at the end, the effective spring constant, *k*, can be related to flexural rigidity by *k* = 3*EI*/*L* ^3^. Combining these two expressions and substituting into (8) allow *L*_*P*_ to be calculated directly from the motion of the tethered Qdot relative to actin (*δ*_*MA*_) and the length of the molecule, *L* (see Figure 2):

(9) $$\begin{array}{c} L_{P}=\frac{L^{3}}{3\delta_{MA}^{2}}. \end{array}$$

In addition to being used to measure the flexural stiffness of MyoVa constructs, the effective motions of the distal rod domain were isolated by using the procedure above with the motion of Qdots bound to MyoVa-HMM constructs rather than actin filaments as the baseline of comparison for MyoVa-FL.

**Figure 6:**
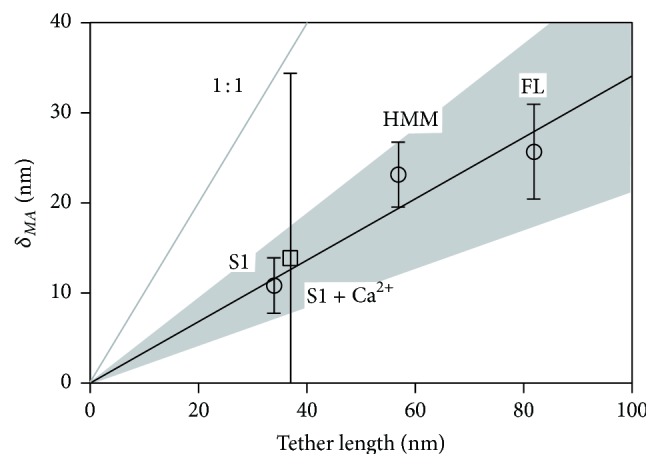
MyoVa construct tethered particle motion relative to actin baseline (*δ*_*MA*_) (open circles, ○) increases with increasing tether length. Solid gray line indicates a theoretical maximum of 1 : 1, where the tethered particle motion equals that of the tether length itself. MyoVa-S1 tethered particle motion in the presence of calcium (square, □). The data were fit with a slope of 0.341 nm/nm, with the 95% confidence interval for the fit shaded in gray.

The validity of the tethered particle motion approach was confirmed by defining the relationship between myosin tethered Qdot motion relative to the actin filament (*δ*_*MA*_) (see (5)) and the assumed tether length, *L*, for the various MyoVa constructs, which was estimated to span from the actin filament surface to the center of the attached 20 nm Qdot, with the length of the MyoVa construct based on its structure [27] (see Figure 2). To test the significance or the trend in *δ*_*MA*_ with *L*, a linear regression was performed using Prism 6 (GraphPad Inc.). In general, *δ*_*MA*_ was expected to increase with increasing *L*, which in fact was the case (see Figure 6). A pilot study showed no significant effect of up to 100 nm of displacement out of the focal plane on Qdot image size parameters (see Figure S1 in Supplementary Material available online at http://dx.doi.org/10.1155/2015/465693), confirming that the trend in increasing *δ*_*MA*_ with *L* was, in fact, the result of tether compliance and not focal separation [34] between tethered and reference Qdots.

## 3. Results and Discussion

To estimate the flexural stiffness of MyoVa-FL and its subdomains, we utilized long exposure time TPM imaging of Qdots attached to various MyoVa constructs that served as flexible tethers (Figure 2). The long exposure times were necessary since the Brownian excursion time for a Qdot tethered to a MyoVa construct should have been on the order of microseconds [22]. Based on our experience, imaging Qdots with acceptable signal : noise was limited to exposure times in the millisecond range. Thus, accurately assigning the tethered Qdot's location at a single point in time was effectively impossible. However, at the other extreme, millisecond exposures do not fully sample the Qdot's excursion overall times. Therefore, to accurately estimate MyoVa's flexural stiffness, the full range of tethered Qdot motion is needed; otherwise the ambiguity in tracking the Qdot's position results in a significant underestimate of motion [23]. Imaging for relatively long exposure times (≫100 ms) by summing sequentially acquired images (see Section 2) (Figure 3) circumvents this issue by sampling the full distribution of particle locations and becomes insensitive to exact exposure times. A further advantage of TPM is the ability to efficiently estimate the flexural stiffness of a large number of MyoVa constructs simultaneously within each visual field.

**Figure 7:**
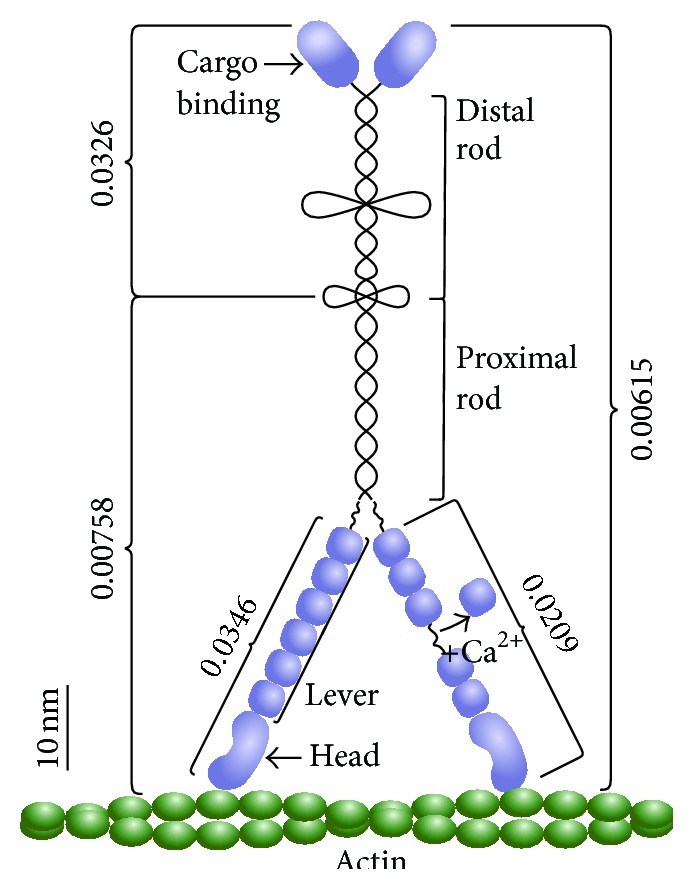
Scale representation of MyoVa with flexural stiffness of various subdomains in pN/nm calculated from (5).

We measured image size parameters for Qdots bound to actin and tethered by various MyoVa constructs. Before relating these parameters to estimates of tether motion and thus flexural stiffness, it was important to control for the effective motion (i.e., noise) in the imaging system, the mode of Qdot attachment, and the actin filament movement to which the various MyoVa constructs were bound (Figure 2). To control for the imaging system noise, image size parameters were obtained for Qdots bound to the microscope slide and the NEM-modified myosin layer under identical imaging conditions (Figure 2). These image size parameters were no different (185.8 ± 2.8 nm and 184.3 ± 2.3 nm mean ± SEM, resp.), indicating that image size parameters for Qdots in the visual field that were not bound to actin and are suitable for use as stationary, surface-bound Qdot references, *S*_*R*_. However, Qdots bound directly to actin filaments showed 17 nm of motion, *δ*_*T*(*A*)_, relative to reference Qdots, *δ*_*R*(*A*)_ (Table 1). This amount of motion indicates substantial compliance in the NEM-modified myosin attachment to the actin filaments, requiring the use of actin-bound Qdots and their motion as a corrective baseline for MyoVa stiffness estimates (Table 1). Using a range of different length MyoVa constructs (Figure 2), we observed a proportional increase in tethered Qdot motion relative to actin, *δ*_*MA*_, with increasing tether length, that is, the length of the MyoVa construct, *L* (Table 1; Figure 6). The linear regression of *δ*_*MA*_ with respect to *L* was statistically significant (slope = 0.341, *p* < 0.01), with all points falling between the theoretical bounds of 1 (which assumes a tether with zero stiffness) and 0 (perfectly rigid tether). In addition, to the flexural stiffness of the various MyoVa constructs, we could estimate the flexural stiffness of MyoVa subdomains, that is, the lever arms, lever arm/coiled-coil junction, and distal rod (Table 1, Figure 7) as presented below.

### 3.1. Lever Arm Stiffness

MyoVa's lever arms and their elasticity [35] are critical to the motor's processive movement [10]. Differential strain in the motor's lever arms while undergoing processive movement is apparent in electron [20] and atomic force [7] microscopic images in which bending of the leading head's lever arm is observed as it attempts its power stroke against the resistive load of the strongly bound trailing head. With the kinetics of the chemomechanical cycle of the individual heads being load-dependent, the difference in strain experienced by the leading and trailing heads is believed to underlie the gating mechanism that assures forward displacement of the motor along actin [21, 36]. Therefore, the lever arm flexural stiffness may be tuned via calcium signaling to effectively allow or inhibit this necessary intramolecular communication between heads.

Using TPM, the motion of Qdots attached to the C-termini of single-headed MyoVa-S1 constructs relative to actin-bound baseline Qdots was used to estimate the lever arm stiffness (see (5)). Based on electron microscopic images, the position of a strongly bound MyoVa head domain appears fixed on the actin filament relative to the observed swinging motion of the lever arm during the power stroke [20]. Therefore, we assumed that the tethered Qdot motion predominantly reflects the 0.0346 pN/nm flexural stiffness of the lever arm (Figure 7; Table 1) with a corresponding persistence length of 242 nm. A significantly higher stiffness value of 0.21 pN/nm for the MyoVa-S1 was estimated using oscillatory length perturbations in the laser trap assay [36]. However, this approach most likely imposed a combination of bending and axial tension, which would be expected to lead to higher stiffness values. The TPM method more closely approximates pure bending, so that the TPM estimate for persistence length agrees with our previous estimate of 310 nm, based on MyoVa-S1 step size measurements in a laser trap assay [29]. In these earlier studies, the motor itself imposed an internal bending strain on the lever arm due to the power stroke working against the stiffness of the laser trap and thus would have been dominated by bending rather than tensile forces.

Following the addition of calcium, the average motion of Qdots tethered to the MyoVa-S1 C-terminus increased by 10%, indicating a 40% reduction in lever arm flexural stiffness (Figure 7; Table 1). A substantial decrease in stiffness is expected, given that the addition of calcium can disassociate as many as three calmodulins from each lever arm, exposing the underlying alpha-helix, which by itself is assumed to be unstable [12, 13, 37]. It is important to note that although calmodulins could dissociate from the lever arm in the absence of calcium without excess calmodulin in the assay buffer, as in our experimental conditions, we do not believe this was the case. It has been shown that MyoVa-HMM processivity, as defined by the motor's run length, is a sensitive measure of interhead communication that relies on rigid lever arms [12, 37]. Thus, the addition of calcium leads to an abrupt decline in processivity as calmodulins dissociate and the lever arms become compliant [12, 37]. However, in control experiments (Figure S4 in Supplemental Materials), run lengths for MyoVa-HMM with and without excess calmodulin in the assay buffer were unchanged, confirming that the lever arms' calmodulin occupancy under our zero calcium conditions was fully intact. Interestingly, in addition to an increase in the average tethered Qdot motion with calcium, there was a large increase in the variance of the Qdot motion, with the SEM increasing from ±3 nm to ±21 nm (Figure 6). The number of calmodulins dissociated from each individual lever arm is variable, with an average of 2.2 per lever [13, 37], which may account for the broad distribution of observed tethered Qdot motions for the MyoVa-S1 construct in the presence of calcium (Figure 6; Table 1). The observed decrease in lever arm flexural stiffness is likely large enough to negatively impact the interhead communication and gating [38] necessary for force production and processivity [12].

### 3.2. MyoVa Rod Stiffness

The MyoVa rod domain lies between the lever arms and the globular tail domain (Figure 7). This rod is predominantly *α*-helical coiled-coil and contains two PEST sites, with the *α*-helical coiled-coil in the proximal rod prior to the first PEST site necessary for MyoVa dimerization [39] (Figure 7). To estimate the flexural stiffness of the distal rod domain, we compared the tethered Qdot motion of the MyoVa-FL construct to that of the MyoVa-HMM construct, which was truncated just before the first PEST site (Figures 2 and 7). Our measurements suggest that the distal rod segment, itself, has a flexural stiffness of 0.0326 pN/nm. Unlike the lever arm, however, it is difficult to apply (9) and calculate a persistence length for the rod segment, as the exact length and structure of the distal rod are unknown, with estimates of the distal rod ranging up to 38 nm not including the 7 nm globular tail domain [4, 27]. The observed increase in tethered particle excursion requires the distal rod to have some effective length, though the measured stiffness requires a structure composed of either a long (~35 nm) segment of coiled-coil (*L*_*P*_ = 100 nm [35]) or a series of coiled-coil segments interspersed with highly compliant disordered segments. Both the regulatory interaction between the globular tail and motor domains [27, 40, 41] and the variation in apparent rod lengths in electron micrographs strongly suggest the latter.

Interestingly, the flexural stiffness for the MyoVa-HMM and MyoVa-FL constructs is an order of magnitude lower than both the distal coiled-coil rod domain and lever arms (Table 1, Figure 7). With the TPM measurements for both of the MyoVa-HMM and MyoVa-FL constructs obtained in rigor, we assume that for either construct both heads are strongly bound to the actin filament [8]. The two-headed bound state with the two lever arms in a triangular arrangement [20] should give a combined stiffness for the two levers, which is greater than a single lever arm and greater than the flexural stiffness of the rod. Thus, the extremely low MyoVa-HMM and MyoVa-FL flexural stiffness require that the lever arm/rod junction must be highly compliant. This compliance must contribute to the free diffusive search of the unbound leading head [18, 19], allowing MyoVa to maneuver through actin-actin intersections [14] and to switch tracks on actin bundles [15].

## 4. Conclusions

We have developed a unique system, utilizing long exposure TPM, to measure the flexural stiffness of single molecules with lengths less than 100 nm. The estimates of flexural stiffness for various MyoVa constructs and subdomains by TPM offer direct insight into the structural components of the MyoVa motor that are critical to its functional capacities. Specifically, the lever arms must be rigid enough to allow intramolecular strains to be communicated between heads for proper gating and control of the motor's processivity. The compliance of the lever arm/rod junction must have sufficient flexibility to allow MyoVa to avoid intracellular obstacles and deliver cargo efficiently to its destination in vivo through the dense actin meshwork. In addition, the potential for alterations in the lever arm stiffness with changes in intracellular calcium may provide a measure of tunability in regulating cargo transport and delivery linked to changes in the cell's ionic environment.

## Supplementary Material

 Supplemental material contains additional validation tests for the tethered particle motion technique. Measurement of quantum dot image size as a function of focus distance confirmed that tether length itself did not bias size measurements. Size parameters for single and paired Qdots were compared in order to verify that pairs of Qdots appear much larger than either stationary Qdots or Qdots moving as a result of tether deformation. Stationary Qdots were also used to confirm that measured image size is independent of the location of a Qdot on our microscope's field of view. Additionally, processivity of MyoVa motors on actin filaments was used to confirm (by way of run length measurement in the presence or absence of excess calmodulin) that in our S1 experiment (without added calcium), calmodulin occupancy was at saturation.

## Figures and Tables

**Table 1:** Calculated tethered motions and mechanical properties of MyoVa subdomains. The tethered motions and mechanical properties ± propagated standard errors are derived from image size parameters for the tethered Qdot (*S*_*T*_) and reference Qdot (*S*_*R*_) (see Section 2). The numbers of measured particles in the tethered (*n*_*T*_) and reference states (*n*_*R*_) for each domain are presented. The tether lengths (*L*) are those presented in Figure 2. Persistence length is given for the S1 subdomain but is not applicable for more complex geometries.

Subdomain	*n*_*T*_	*n*_*R*_	Tether length, *L* (nm)	Motion versus reference, *δ*_*T*_ (nm)	Motion versus Actin, *δ*_*MA*_ (nm)	Flexural stiffness, *k* (pN/nm)	Persistence length, *L*_*P*_ (nm)
Actin	888	69	NA	17.38 ± 5.56	NA	NA	NA
S1	393	66	44	20.47 ± 15.80	10.82 ± 5.92	0.0346 ± 0.0379	242 ± 109
S1 + Ca	98	9	44	22.69 ± 19.94	13.92 ± 22.25	0.0209 ± 0.0669	146 ± 99
HMM	361	57	67	28.94 ± 11.35	23.14 ± 3.61	0.00758 ± 0.00236	NA
FL	327	69	92	31.01 ± 9.27	25.69 ± 5.24	0.00615 ± 0.00251	NA
Distal rod	NA	NA		NA	11.15 ± 4.82^*∗*^	0.0326 ± 0.00282	

^*∗*^Distal rod deflection was calculated from FL relative to HMM rather than Actin.
