# Supplementary material for: Flexural Stiffness of Myosin Va Subdomains as Measured from Tethered Particle Motion
Source: J Biophys. 2015 Nov 30;2015:465693. doi: 10.1155/2015/465693 (PMC4685436; doi:10.1155/2015/465693)

Supplemental Material

Image Size Parameter Controls

One potential limitation of the TPM technique is the increase in Qdot point spread size with distance outside of the focal plane, which could potentially bias calculations of relative motion. Since the microscope was focused manually, and the majority of Qdots were tethered, this bias would lead to an underestimate of tethered particle motion. To address this concern, a validation study was performed using a nano-positioning piezo stage to image glass bound Qdots at various amounts of de-focus. Six fields of view with nine to twelve glass bound Qdots each were imaged in TIRF. The nano-positioning stage was moved in the z-direction in discrete 25nm steps from 0 to 100nm and back, and an image was acquired at each step. Following acquisition, the image size parameter of each Qdot was measured using the same routines used for myoVa tethered Qdots. Mean image size parameters for each field of view were then plotted versus z-distance in Prism (Graphpad Inc), and linear regressions were performed (see Figure S1). The slope of the linear fit was not significantly different from zero (p-values ranged from 0.1182 to 0.7517) for any of the six fields of view. Within the maximum length of proteins used in this study (100nm) of the focal plane, the apparent size of glass bound Qdots varied by only 1%.

Additional factors potentially affecting the distribution of image size parameters include aggregation of labeled proteins and variance across the camera's field of view. The effect of protein aggregation was tested by preparing a 1:1 mixture of biotin and streptavidin conjugated Qdots. As each streptavidin may bind up to four biotins, this sample contained single Qdots along with aggregates of up to five. A total of N points met our criteria for image size analysis (Figure S2). In addition to a large peak at 160nm, a second peak emerged at 215nm, in contrast to the smooth distribution yielded by images of Qdot labeled MyoVa (Figure 5). The 215nm peak most likely represents paired Qdots, while larger aggregates fell outside of our criteria for analysis.

In order to confirm that optical aberrations or variation in camera sensitivity did not bias image size parameters, a series of images were acquired of Qdots nonspecifically adhered to a glass coverslip. Figure S3A shows a maximum intensity composite image of 11 fields of view containing a total of 1001 spots. Figure S3B shows the 802 spots meeting our inclusion criteria colored by image size parameter in pixels. No discernable trend was observed with either horizontal, vertical, or radial (distance from center) position (Figure 34B). It is thus expected that

neither optical aberrations nor variations in camera sensitivity systematically biased our measurement of point spread sizes.

Processivity of myoVa-HMM in the presence and absence of excess calmodulin

We have examined if the addition of excess calmodulin to the motility buffer affects the run length of MyoVa-HMM coexpressed with wild-type calmodulin. The motor domain of single MyoVa-HMM molecules were labeled with quantum dots (Qdots) using biotin-streptavidin conjugation and the processive motion of individual MyoVa-HMM molecules observed at saturating ATP [2mM] using an objective-type total internal reflectance microscopy [1]. The run length of individual MyoVa-HMM were measured as described previously [1] with and without excess calmodulin (5 mg/ml) in the assay buffer. The run length histograms were fit to an exponential decay (Fig. S4), providing a run length constant of 982 ±202 nm (with excess calmodulin) and 943 ± 166 nm (without excess calmodulin). These run length constants were not statistically different (p=0.98) as determined using the Kolmogorov–Smirnov Test [2].

References:

1. Ali, M.Y., et al., *Myosin Va maneuvers through actin intersections and diffuses along microtubules.* Proc Natl Acad Sci U S A, 2007. **104**(11): p. 4332-6.

2. Press WH, Teukolsky SA, Vetterling WT, Flannery BP (1992) in *Numerical Recipes in C: The Art of Scientific Computing* (Cambridge Univ Press, Cambridge, UK), pp 623–626.

Figure S1: Mean±SEM image size parameter versus distance in the z-directions, with dashed lines indicating linear regressions for each of six fields of view.

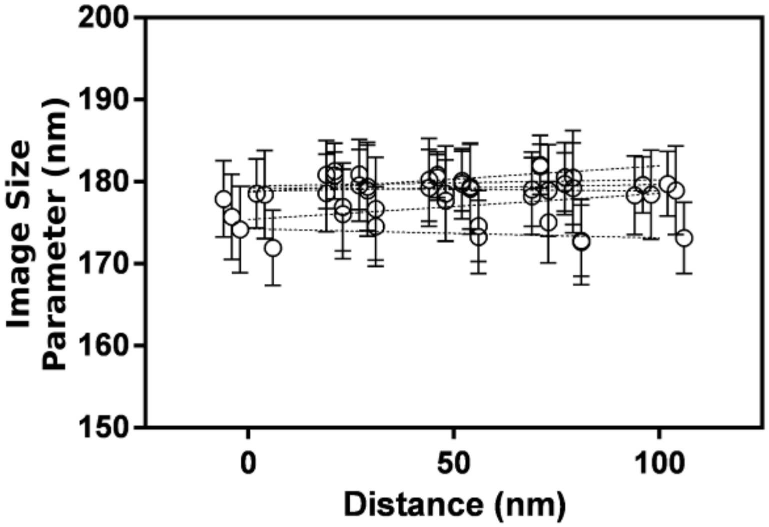

Figure S2: Image size parameter distribution for a 1:1 mixture of biotin and streptavidin conjugated Qdots.

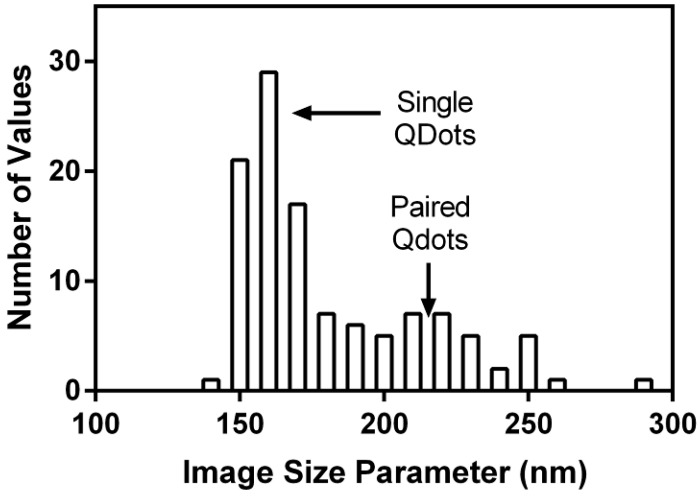

Figure S3: Composite image of fields of surface bound Qdots (A). Spots meeting inclusion criteria colored by image size parameter (B). Image size parameters plotted versus horizontal, vertical, and radial positions (C).

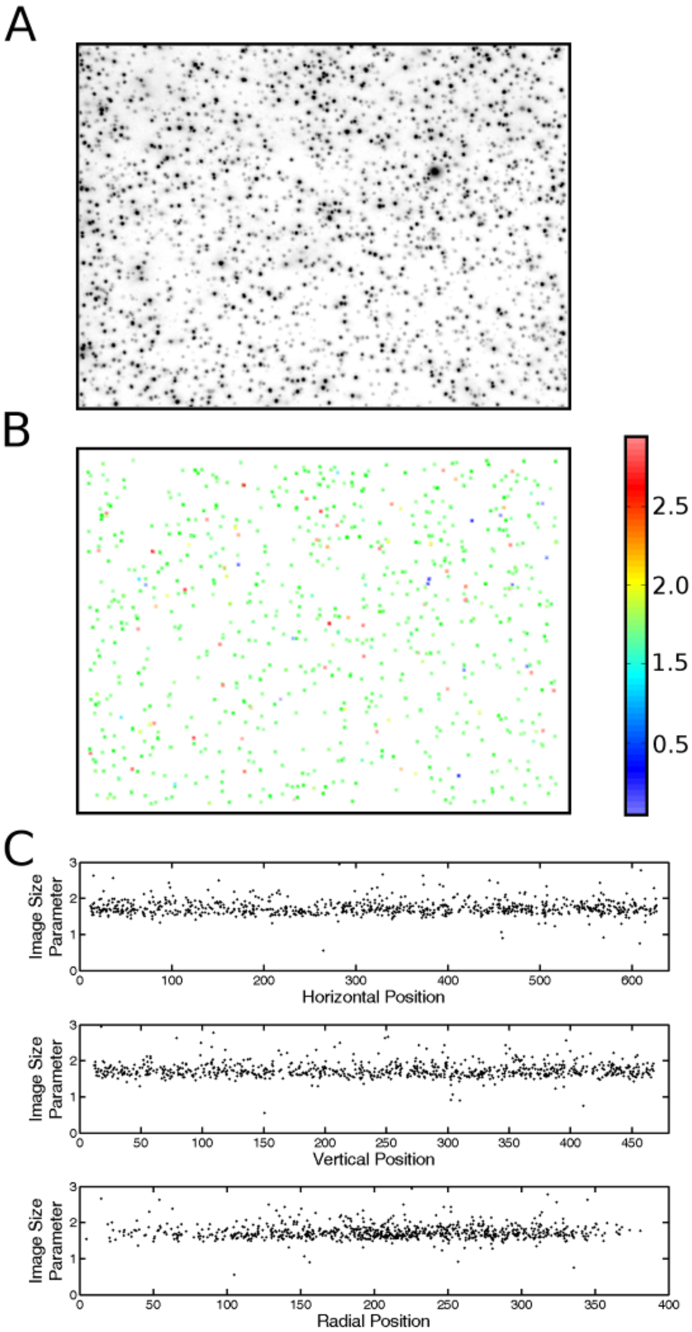

Figure S4: Run length distributions of MyoVa-HMM in the presence (black circle) and absence (red triangle) of calmodulin.

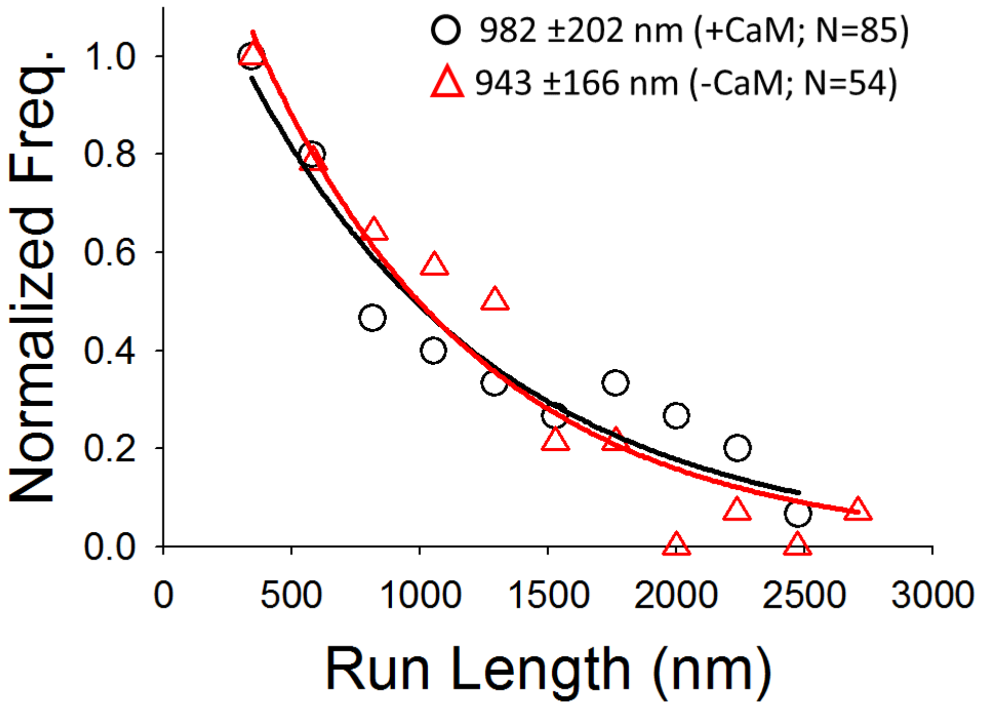

Supplement: Supplementary file 1 — Supplemental material contains additional validation tests for the tethered particle motion technique. Measurement of quantum dot image size as a function of focus distance confirmed that tether length itself did not bias size measurements. Size parameters for single and paired Qdots were compared in order to verify that pairs of Qdots appear much larger than either stationary Qdots or Qdots moving as a result of tether deformation. Stationary Qdots were also used to confirm that measured image size is independent of the location of a Qdot on our microscope's field of view. Additionally, processivity of MyoVa motors on actin filaments was used to confirm (by way of run length measurement in the presence or absence of excess calmodulin) that in our S1 experiment (without added calcium), calmodulin occupancy was at saturation. [file 465693.f1.pdf]
